# Erroneous Vehicle Velocity Estimation Correction Using Anisotropic Magnetoresistive (AMR) Sensors

**DOI:** 10.3390/s22218269

**Published:** 2022-10-28

**Authors:** Donatas Miklusis, Vytautas Markevicius, Dangirutis Navikas, Mantas Ambraziunas, Mindaugas Cepenas, Algimantas Valinevicius, Mindaugas Zilys, Krzysztof Okarma, Inigo Cuinas, Darius Andriukaitis

**Affiliations:** 1 Department of Electronics Engineering, Kaunas University of Technology, Studentu St. 50-438, 51368 Kaunas, Lithuania; 2 Department of Signal Processing and Multimedia Engineering, West Pomeranian University of Technology in Szczecin, 70313 Szczecin, Poland; 3 Communications-atlanTTic Research Center, Department of Signal Theory, University of Vigo, 36310 Vigo, Spain

**Keywords:** traffic intelligent transportation system, magnetic field sensors, AMR, cross-correlation

## Abstract

Magnetic field sensors installed in the road infrastructure can be used for autonomous traffic flow parametrization. Although the main goal of such a measuring system is the recognition of the class of vehicle and classification, velocity is the essential parameter for further calculation and it must be estimated with high reliability. In-field test campaigns, during actual traffic conditions, showed that commonly accepted velocity estimation methods occasionally produce highly erroneous results. For anomaly detection, we propose a criterion and two different correction algorithms. Non-linear signal rescaling and time-based segmentation algorithms are presented and compared for faulty result mitigation. The first one consists of suppressing the highly distorted signal peaks and looking for the best match with cross-correlation. The second approach relies on signals segmentation according to the feature points and multiple cross-correlation comparisons. The proposed two algorithms are evaluated with a dataset of over 300 magnetic signatures of a vehicle from unconstraint traffic conditions. Results show that the proposed criteria highlight all greatly faulty results and that the correction algorithms reduce the maximum error by twofold, but due to the increased mean error, mitigation technics shall be used explicitly with distorted signals.

## 1. Introduction

Recent technological advances have revolutionized intelligent transportation systems (ITSs) in terms of data collection, traffic management, and control. Information obtained by ITSs is necessary to provide numerous services [1,2,3] that enable smoother, safer, and more environmentally friendly transportation. This includes the faster collection of road fees, monitoring the speed of vehicles, and reducing traffic in cities, which result in reduced travel time and the possibility of vehicle accidents. Because of this reason, ITSs are among the viable solutions to help in reaching the goal of the European Union to reduce air pollution by 55% until the year 2030 compared to 1990 [4].

Scientists have investigated various vehicle detection methods, ranging from inductive loops, piezo/quartz [5,6,7], and air switches/road tubes to different magnetometers, microwave radars [8], passive infrared [9], ultrasonic sensors [10], and video surveillance systems [4,11,12]. Proposed methods use either machine learning or feature extraction for the realization of solution algorithms [13]. Testing is completed by either making a prototype system and implementing it, or simulating it with already gathered data from scientists’ older experiments or public databases.

The best known ITSs for traffic classification are those with surveillance cameras. There are even public challenges, which require completing a task using a large number of collected traffic photos. Less common are systems that use passive sensors such as magnetometers [14], accelerometers, or microphones. Comprehensive surveys of sensing technologies for road traffic monitoring are presented in [15,16,17]. The authors agree that camera-based stations are costly, require maintenance, and cannot be powered by batteries. Efficient use of video analysis methods may also be limited by weather and lighting conditions. In many popular applications, such as parking entrances or automatic monitoring of road payments based on register plate number recognition, the controlled lighting may be easily ensured as well as the independence of weather conditions, e.g., under the roof. In addition, due to the growing privacy concerns among the population, video surveillance systems could be more intrusive. Nevertheless, the use of solutions based on magnetic sensors makes it possible to develop a more robust system in open spaces such as a motorway or a city street. Passive sensors are cheaper than video cameras and require less computing power, it allows the development of a distributed monitoring network with low installation expense. On the other hand, ITSs based on low-power passive sensors such as magnetometers, microphones [18], or accelerometers [19], are more challenging to adapt for vehicle classification [20].

## 2. Related Works: Comparison of Speed Estimation Results

Automatic vehicle classification is one of the most applicable features of Intelligent Transportation Systems. It allows for road access control, automatic toll assessment, road planning, pollution estimation, and traffic modeling. Although vehicle velocities are not the main interest, for many systems it is the necessary parameter for length estimation and further classification.

For magnetometer-based monitoring stations, there are two main approaches to estimate the vehicle’s velocity. The first approach is the average speed estimation based on vehicle presence detection and travel time calculation between two longitudinally positioned nodes [21]. The second approach is close to instantaneous speed estimation from a pair of time series signals.

There are several proposals in the literature that are summarized in Table 1. A method proposed by B. Coifman and S. Kim [22] is to use a single-loop detector. Typical traffic speed is estimated within samples of many vehicles, therefore individual vehicle speeds might be far from the typical.

W. Balid [23] explored a vehicle velocity estimation method by two synchronized magnetometer nodes. As it is shown in Table 1, the mean absolute percentage error (MAPE) with sensors, spaced 6 m apart, is 5.1%. As main error factors, the author identifies sampling ratio, synchronization error, and vehicle detection algorithm. 

An interesting region-based technic was suggested by D. Kim et al. [24]. The aim of this algorithm is to clip a region of a pair of magnetic signals and perform cross-correlation calculations to estimate the delay between two time series. Since only up to 14 sample points are used, it requires minimal computation power. For result validation, field tests were performed with two sensor nodes spaced from 1.5 m to 6 m. A dataset of over five thousand samples was collected with vehicles moving at speeds from 26 km/h to 123 km/h. Authors claim that their region-based approach with 6 m spaced sensors estimates speed with an average error of only 1.2%.

Zusheng Zhang et al. [25] proposed a vehicle velocities estimation algorithm immune to electric railway interference. Speed estimation is based on measuring the time gap between the arrival moments of two sensor nodes spaced 8 m apart. As it is stated, the main difficulty is to extract a magnetic signature from a noisy signal and reidentify the same vehicle. The authors performed a field test with constrained and unconstrained driving conditions (drivers could or could not change traffic lanes) and a maximum speed of up to 55 km/h. It was shown that the proposed anti-interference detection algorithm (AIDA) was outperforming other methods with MAPE of 7.5% in unconstrained and 2.6% in constrained traffic conditions.

Kwon et al. [26] used only a 20 cm spaced sensor pair for speed estimation. The time delay was calculated by modified cross-correlation function and adaptive signal rescaling. Field results showed MAPE of 2.1% for vehicles at velocities up to 70 km/h.

A study by Taghvaeeyan et al. [27] shows a 2.5% speed estimation error with 0.9 m spaced sensors at velocities up to 96 km/h. The authors used a cross-correlation algorithm for delay calculation and discrete Fourier transform to reduce the number of operations. Similar results were also shown in [28]; a maximum speed error of 7.5%, and an average of 5% at velocities up to 50 km/h.

As it is seen from the literature review, the spacing of a pair of sensors is the key parameter for estimated speed accuracy. Studies show that simple threshold detection methods provide results with errors of less than 3%, using distances around 6 m between sensors. On the other hand, to have a more compact sensor hub, a cross-correlation algorithm is preferred. In our previous studies [17,29], we demonstrated a speed estimation system with 0.3 m spaced sensors and MAPE below 3%. Even though for most of the applications this error margin is more than enough, it was noted that under certain conditions the estimated velocity result is highly erroneous. Therefore, in this paper, faulty result identification and mitigation algorithms are presented.

## 3. Problem Definition and Experimental Set-Up

### 3.1. Experimental Set-Up

**Figure 1:**
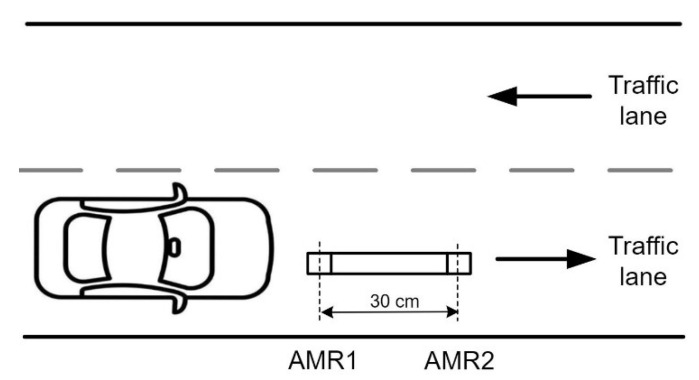
Experimental set-up functional view.

The utilized hardware is comprehensively described in our previous works [17,29]. As shown in Figure 1, the system is composed of two laterally spaced anisotropic resistance magnetometer (AMR) sensors. AMR sensors are deployed with 30 cm spacing and they provide information to the data collection hub via RS485 cable. The sensing hub was installed into the single-lane intercity road with a speed limit of 90 km/h. During a half-year period, a dataset was gathered and then used in the research. Since the road has no physical barriers, drivers are free to move into the sensing area without restrictions.

### 3.2. Erroneous Data Handling

**Figure 2:**
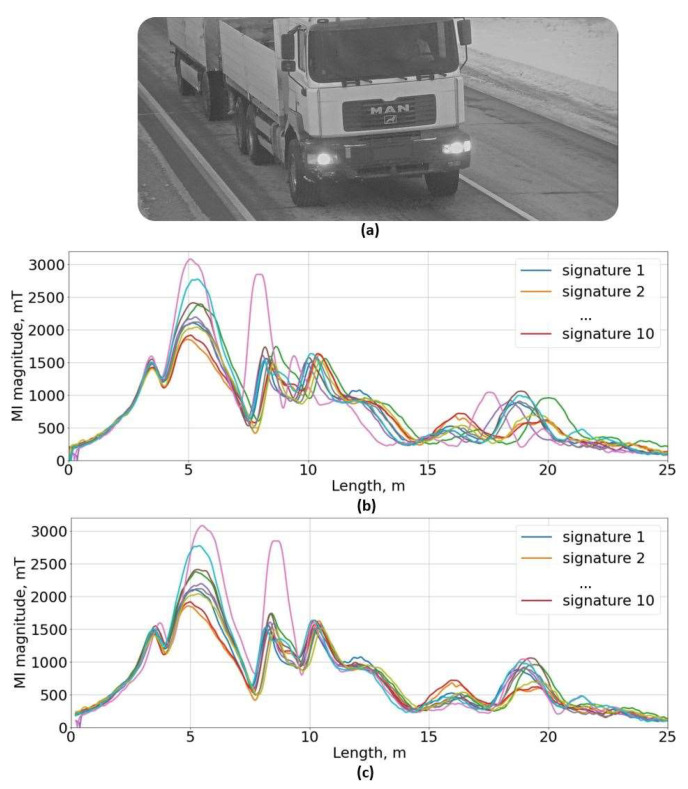
Multiple 18-m truck (**a**) magnetic induction (MI) magnitudes (each color represents unique detection) versus length. (**b**) signatures length estimated according to the speed value from the cross-correlation algorithm, (**c**) corrected signature length by adjusting speed values.

Magnetic field sensors provide feature-rich signals of passing vehicles, but those signals are dependent on multiple factors (e.g., vehicle height, acceleration, and driving trajectory). It has been shown in previously conducted research [27,28,29,30] that cross-correlation is the most accurate method for vehicle speed estimation from a pair of magnetic signatures. In Figure 2b, multiple magnetic signatures of the same vehicle are shown versus the length. Although it is the same vehicle, the signature features points do not match due to wrongly estimated velocity. To match all signals, the speed for each detection had to be adjusted from −7.5% to 12%. Due to the finite sampling frequency and average traffic velocities of 80 km/h, it is expected to have a speed error of up to 5%. However, during the actual traffic conditions, errors are twice as high than in theoretical estimation and this is probably related to the distortions in a pair of magnetic signatures. Due to asymmetric features, the cross-correlation algorithm is not able to align two signals correctly.

## 4. Methods and Materials

Using only the signals from the AMR sensing hub, it is not a trivial task to judge whether the estimated vehicle velocity value is correct or faulty. Although there is no single characteristic of faulty results, mostly the reason of high errors are asymmetrical pair of signals. After an analysis of the related works, we could not find any alternative solution for erroneous results mitigation. Therefore, authors propose and validate a criterion of cross-correlation significance and velocity correction algorithms.

### 4.1. Cross-Correlation Significance Estimation

**Figure 3:**
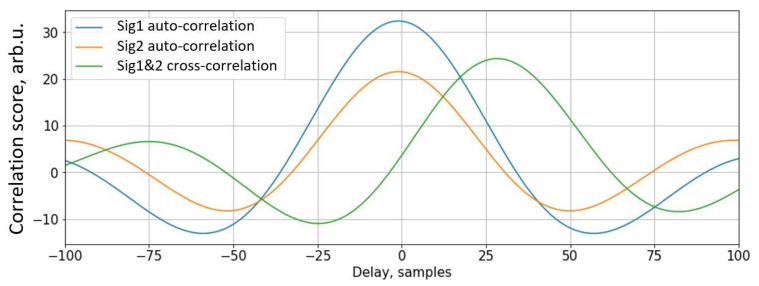
Comparison of auto and cross-correlation functions for a pair of magnetic signatures (arb. = arbitrary units).

Estimated velocity result significance can be verified by comparing peak values of correlation results. In Figure 3, a cross-correlation and auto-correlations of a pair of magnetic signatures are shown. As it is visible, the pair of signals are not identical and the auto-correlation of the first signal is much higher. In theory, for two identical shapes but different level signals, the peak of cross-correlation should be the arithmetic mean of auto-correlations peaks. Consecutively, for a pair of asymmetrical shapes, this value is lower. 

A cross-correlation significance coefficient is calculated as follows:

(1) $$K=\frac{mean\left[peak_{ac1}:peak_{ac2}\right]-peak_{cc}}{mean\left[peak_{ac1}:peak_{ac2}\right]},$$

where *K* is the significance coefficient, *peak_ac*1*_* and *peak_ac*2*_* are the peak values of auto-correlation functions for pair of magnetic signatures and *peak_cc_* is the peak value of the cross-correlation function.

**Figure 4:**
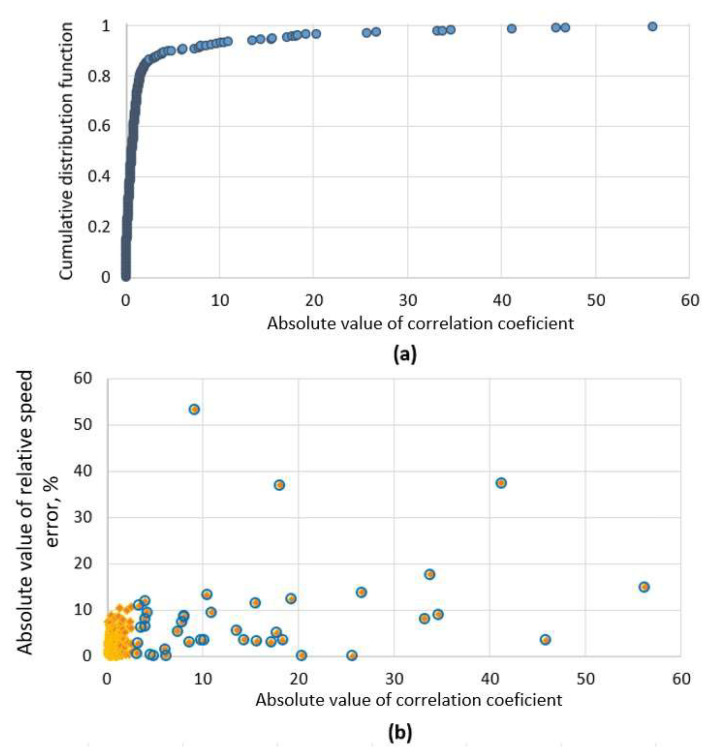
Top (**a**): cumulative distribution function of correlation significance coefficient for a dataset of 300 cases. Bottom (**b**): absolute value of relative speed error versus correlation significance coefficient. Cases with highly distorted signals are marked with blue circles, while regular signatures are orange.

The proposed criterion was tested with a dataset of 300 unique magnetic vehicle signatures recorded during normal traffic conditions and plotted in Figure 4a. As it is visible, 90% of all signatures in the dataset estimated coefficient value is below five, which shows a high similarity between the two signals. On the other hand, in Figure 4b, it is seen that high-speed errors (above ±10%) are common only in cases with a high correlation significance coefficient. The coefficient helps to indicate which signatures are distorted, although it does not mean that the estimated velocity is wrong. On the other hand, based on this information, we can label potentially erroneous cases and perform different velocity estimation algorithms.

### 4.2. Non-Linear Peak Suppression

It is common that a magnetic signature has few significant peaks and this is especially common for distorted signatures. Since the cross-correlation result depends on signal amplitude value, peaks have a higher influence than valleys.

**Figure 5:**
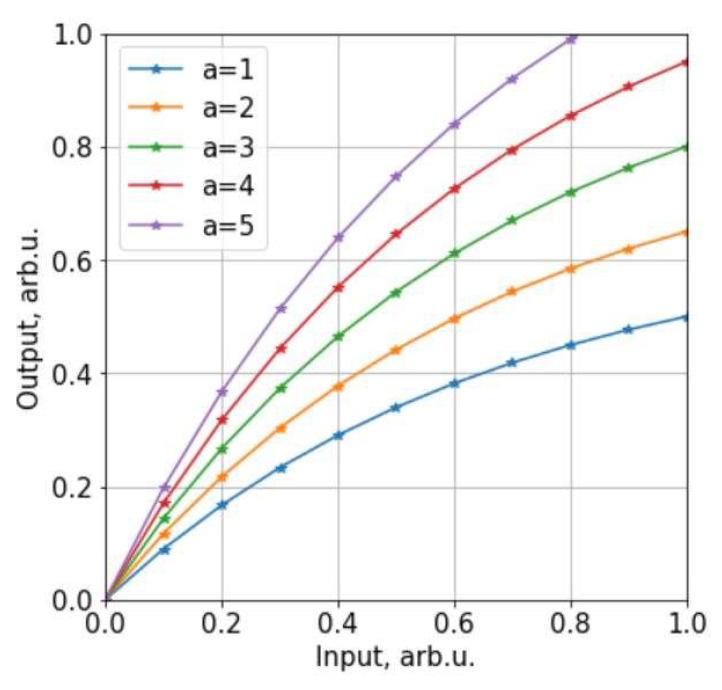
Non-linear scaling function with different coefficient values (arb.u. = arbitrary units).

It was proposed to apply non-linear signal scaling to suppress peak influence for signal alignment. Firstly, signals were normalized by mean value and then scaled according to Formula (2) (Figure 5).

(2) $$F_{d}=1-e^{-aF},$$

where: $F_{d}$ is the rescaled value; F, the original value; and a, a rescaling coefficient.

**Figure 6:**
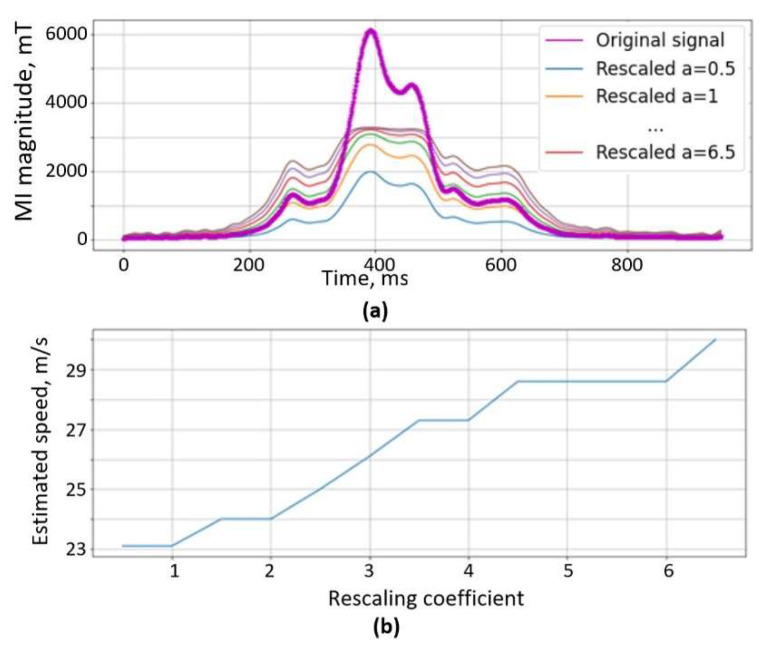
(**a**) Original signal (purple) with rescaled signals, (**b**) speed versus *a* coefficient.

In Figure 6a, the original (purple) signal is plotted with rescaled signals, once peaks have been suppressed. In Figure 6b, estimated velocities are plotted versus rescaling coefficient a. As is visible in the plots, by increasing suppression coefficient *a*, the estimated speed value increases. For this particular case, the ground truth speed value was 26 m/s and this value was achieved with the rescale coefficient *a* = 3. Although every pair of signals is a unique case and requires different coefficient *a* value for the lowest error, our aim is to tune the algorithm to filter out highly erroneous cases. The algorithm is validated with the dataset in the results section.

### 4.3. Regional Signals Correction Algorithm

Another author’s proposed approach is to locate distorted regions in the pair of signatures and to reduce their weight on signal alignment. From Algorithm 1, initially, a pair of magnetic signals are aligned according to the average traffic velocity. Then, the Euclidian distance vector (*diff*) is calculated and the interquartile range is estimated. It gives a rough estimation of the standard distance between two signals, while much larger values show distorted regions. These regions are clipped if values exceed interquartile range values and then, corrected magnetic signatures are constructed.
**Algorithm 1:** regional signals correction

1:  **align**
*modul1* and *modul2* by average traffic speed

2:  *diff* = *modul1* − *modul2*

3:  [Q1, Q3] = interquartile (*diff*)

4:  **if** (*diff*_(***i***)_ < Q1 **or**
*diff*_(***i***)_ > Q3) **then**

5:   *diff*_(***i***)_ = [Q1 **or** Q3]

6:  **end if**

7:  *corrected_modul1 = modul1* − *diff*

8:  *speed* = 
$\frac{sensors\_gap}{cross\_correlation\left(corrected\_modul1,\;\;\;modul2\right)\;}$

9:  **result**
*speed*


**Figure 7:**
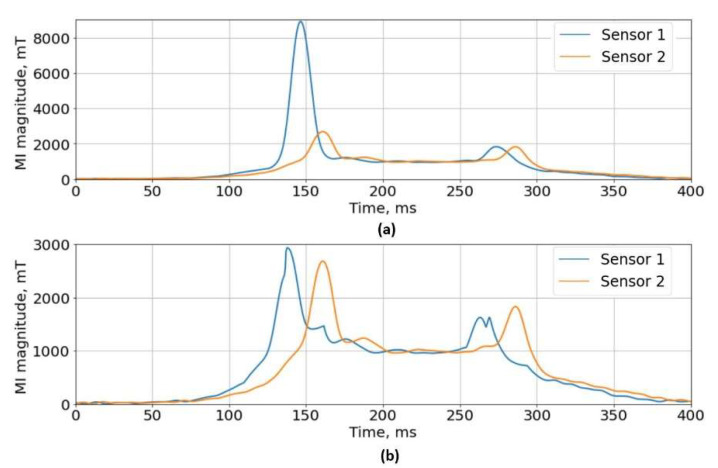
(**a**) Original pair of magnetic signatures, (**b**) corrected pair of signatures.

An example of inputs and processing results are shown in Figure 7. Two signals were aligned by the average traffic velocity (Figure 7a) and only the highly mismatched regions were corrected by subtracting the difference (Figure 7b). It is clear that the initial alignment step is very important and for vehicles driving at a very different speed than the average traffic—this algorithm is not appropriate.

### 4.4. Signals Segmentation Algorithm

Inspired by modified cross-correlation algorithms from [24,26] it was proposed to utilize magnetic signature segmentation in order to isolate distorted features. Signal segmentation is performed on the first order derivative of the signature since it crosses the zero level multiple times. In this manner, sub-signals are obtained, and cross-correlation is performed separately to each sub-pair of signals to estimate the delay, and eventually the speed value. A detailed view of the calculation steps is shown in Algorithm 2, where *modul1*(2)—vector of magnetic signature magnitude, *deriv1(2)*—vector of difference quotient of magnetic signature, zeros1(2)—vector of zero crossing points and *S_LIST*—list of estimated speeds.
**Algorithm 2:** Signals segmentation

1:  *deriv1(2)* = derivative (*modul1(2)*)

2:  *zeros1(2)* = zero_crossing (*deriv1(2)*)

3:  **for** 
(i=0; i 
≤
length(zeros1);
i++)

4:   *sub1* = *modul1*[ 0:zeros1[
i] ]

5:   *sub2* = *modul2*[ 0:zeros2[
i] ]

6:   *speed* = 
$\frac{sensors\_gap}{cross\_correlation\left(sub1,\;\;\;sub2\right)}$

7:   add *speed* into *S_LIST*

8: **end for**

9: **for** 
(i=
length(zeros1); 
i 
**≥**
** 0; i−−**)

10:   sub1 = *modul1*[ zeros1[i]:end ]

11:   sub2 = *modul2*[ zeros2[i]:end ]

12:   *speed* =
$\frac{sensors\_gap}{cross\_correlation\left(sub1,\;\;\;sub2\right)}$

13: add *speed* into *S_LIST*

14: **end for**

15: **result**
*S_LIST*


**Figure 8:**
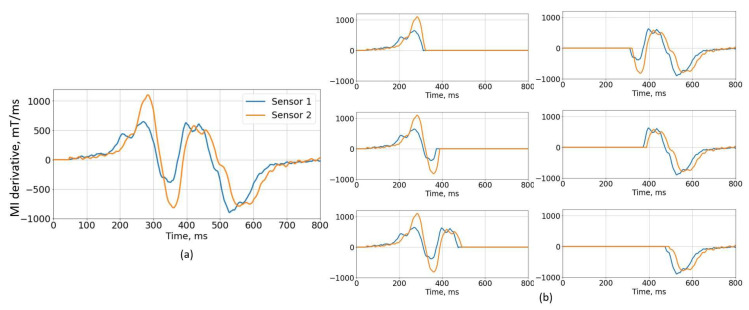
Signals segmentation algorithm example. (**a**) The first order derivative of the signatures is divided into 6 sub-signal pairs (**b**).

The principles of the algorithm are depicted in Figure 8. Both signals (Figure 8a) cross the zero level three times. Therefore six (Figure 8b) new sub-pairs are obtained and speed values from 24 m/s to 42.7 m/s are acquired. Visually, it is clear that the first half of the signal is highly distorted and shall not be used. Using only the second part of the signal, we obtained speed values from 24 m/s to 31 m/s, while the actual value was 24 m/s.

## 5. Results and Discussion

### 5.1. Non-Linear Signal Suppression Correction Results

As it is stated in the related work section, speed estimation using a cross-correlation algorithm gives an average error from 1.2% to 2.8%. However, with distorted signals, the estimated value might spike up to two or three times higher than the actual speed. 

Results of non-linear signal rescaling are shown in Table 2, involving two test groups analyzed under five different calculation approaches. As a reference for velocity and length calculations, a piezoelectric sensor hub was used to measure vehicle’s ground-truth velocities while the length values were obtained from car manufacturers. The first test group consists of 300 unique magnetic signatures registered during normal traffic conditions, while the second group consists of 40 highly distorted signals. The first column in the table shows the estimated speed value without correction. In columns (2), (3), and (4), scaling algorithm results with different scaling coefficients are shown. Additionally, in column (4), the first order derivative was used. For the last two columns, additional filtering was applied by a low pass filter of 30 Hz and 10 Hz, which additionally smoothened out the signals.

As it is shown in Table 2 for the regular signature group, the average percentage error and the maximum absolute error is the smallest for the algorithm with unscaled magnetic signatures. On the other hand, for only the distorted signature vehicle group, variable parameters were tested, and the algorithm yields significant maximum error reduction. The peak suppression algorithm helped to reduce average error by two percentage points and importantly, the maximum error was reduced by twofold.

**Figure 9:**
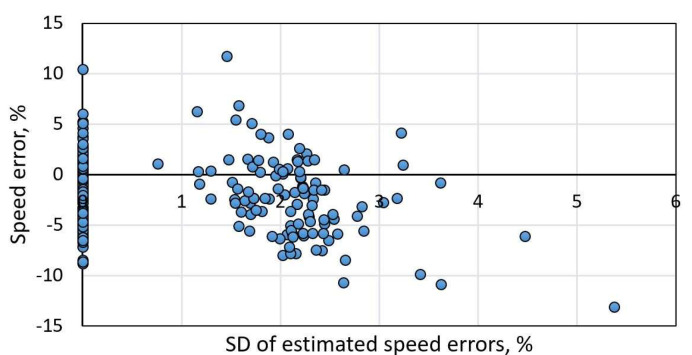
Relative speed error versus standard deviation (SD) of estimated speed error values by applying peak suppression with five different rescaling coefficients.

As an additional to faulty result indication, an estimated results variation was considered. It was expected that estimated speed values should not change for regular signatures by applying a peak suppression algorithm with different scaling coefficients. On the other hand, for the distorted signatures with asymmetric peaks, this technique should lead to significant deviation in results. Figure 9 shows the relationship between estimated speed errors (large errors are related to distorted signatures) versus estimated speed deviation by applying five different rescaling coefficients. As it is visible, even low volatile results might have large speed errors (>10%). Presumably, distorted signals have significant features, which lead to incorrect signal alignment. Although non-linear signal rescaling should suppress the weight of those aspects, it depends on the ratio between “good” and “bad” features. 

Similarly, the regional signal correction algorithm was tested. It was found that with the distorted group of signatures which initial estimated speed error range from −130% to 37% and MAPE 11% after the correction maximum error was reduced to 33% and MAPE 9.5%. The obtained results confirm the initial assumption. The average traffic velocity value might be used to correct the signals and reduce the error range significantly. On the other hand, when the same approach was used with the signatures of regular shape, the average error increased. Therefore, the proposed algorithm shall be used only for potentially distorted cases.

### 5.2. Estimation Results of Signal Segmentation

A test group of 20 vehicles was selected with highly distorted signatures to test the speed estimation algorithm by segmenting the signals. The first order derivatives were calculated for every pair of signals and divided into two parts at the zero crossing point. Consecutively, two velocities values were obtained by applying a cross-correlation. Table 3 contains the estimated speed results from the signature, 1st and 2nd half of the derivative, and the corrected speed error. As it is shown, for cases with highly faulty results (e.g., No. 4), the speed values from segmented signals differ significantly. Therefore, from two values, we selected a lower and more meaningful value (the speed value cannot be negative or too large). For this highly distorted test group, the proposed algorithm reduced MAPE from 21% to 17%, and the maximum error was reduced from 129% to 61%. The approach was also tested with the group of the regular signatures and the results are similar compared to the previously described algorithms i.e., an increase of the average error. Therefore, it is essential to derive reliable erroneous results criteria as shown in Section 4.1, and apply one of the correction algorithms only to highlighted cases.

## 6. Conclusions

In this paper, we address the problem of occasionally high vehicle speed estimation errors, based on the AMR type of magnetic sensors. As explained in the related works section, cross-correlation is the main method for estimating time delay between the two time series. Multiple authors [2,24,25,28] claim that with sufficiently spaced sensors (~6 m), speed can be estimated with an average error of less than 2%. On the other hand, occasionally due to differentiable pairs of signals, a particular method produces highly faulty results.

Three different algorithms were analyzed for identifying and correcting highly erroneous results. Non-linear signals’ peak suppression and regional distortion correction yield similar results. It helped to reduce the maximum estimated speed error by twofold for a distorted signature group. Furthermore, an experimental test showed that signal segmentation into sub-regions helps to avoid high errors, although it adds more complexity. On the other hand, since the analyzed algorithms increase the mean error, they shall not be used blindly for all measurements. Therefore, a criterion to label potentially erroneous cases was also investigated. The proposed cross-correlation significant coefficient identified all distorted signals in our dataset, although even highly distorted signatures might have low estimated speed error.

The AMR magnetic sensors-based vehicle classification system is a viable alternative to classical video surveillance [14,15] or radar systems [11]. Multiple researchers [24,25,26,27] show that with commercially available hardware (e.g., sensors and micro-controllers) it is feasible to create a low-cost and practically valuable monitoring hub. Nevertheless, due to intrinsic traffic dynamics and complex signals, more research is needed to prepare the technology for commercial use. Although our proposed algorithms helped identify and significantly reduce the maximum speed error, particular cases still contain large errors (>50%). It is planned to add an additional accelerometer-based sensing channel to fuse data and increase reliability.

## Figures and Tables

**Table 1:** Speed estimation result comparison with magnetic field sensors.

Author	Sensor Spacing	Speed Error	Description
B. Coifman et al. [22]	Single sensor	Up to 60 km/h	Single-loop detector and speed estimation from a measurement of many vehicles. Moving median speed estimation.
W. Balid et al. [23]	6 m	MAPE 5.1%	System tested on the highway. Speed estimated algorithm based on arrival moment detection (threshold approach).
D-H. Kim et al. [24]	6 m	MAPE 1.2%	Modified cross-correlation algorithm with only region of interest from the signals. System tested in the field with vehicles at velocities from26 km/h to 123 km/h.
Z. Zhang et al. [25]	8 m	MAPE 7.5%	MAPE in unconstrained traffic mode 7.5%—constrained 2.6%. Speed is estimated from the time gap between two vehicle events.
Y. Kwon et al. [26]	0.2 m	MAPE 2.1%	Speed estimated using cross-correlation algorithm. Vehicles at speed up to 70 km/h.
S. Taghvaeeyan et al. [27]	0.9 m	MAPE 2.5%	Speed estimated using cross-correlation and discrete Fourier transform algorithms, vehicles speed up to 96 km/h.
H. Zhu et al. [28]	5 m	MAPE 5%	Speed estimated using cross-correlation algorithm, vehicles speed up to 50 km/h.
V. Markevicius et al. [17,29]	0.3 m	MAPE 2.6%	Vehicles at velocities up to 120 km/h. System tested with all kinds of vehicles in un-constrain traffic conditions.

**Table 2:** Comparison of the performance of rescaled speed estimation algorithms.

	(1) Unscaled sig.	(2) Scaled sig. a = 1.5	(3) Scaled sig. a = 2	(4) Scaled 1st deriv. a = 1.5	(5) Filter LP 30 Hz, a = 1	(6) Filter LP 10 Hz, a = 1
	Regular signature group					
MAPE, %	3.0	3.4	3.2	3.5	3.5	5.6
STD, %	3.5	4.4	4.2	5.5	4.3	8.9
ABS max %	13.1	24.3	24.3	52.0	26.3	75.2
	Distorted signature group					
MAPE, %	11.3	9.8	9.2	28.5	10.2	13.4
STD, %	25.1	15.1	14.3	56.8	17.4	20.8
ABS max %	129.4	57.2	50.0	229.8	63.6	70.6

**Table 3:** Estimated speed correction by signal segmentation.

Test, No#	Actual Speed, m/s	Estimated Speed, m/s			Estimated Speed Error, %			
		Signature	Deri. 1st Half	Deri. 2nd Half	Signature	Deri. 1st Half	Deri. 2nd Half	Corrected Speed Error
1	16.7	10.5	9.5	120.0	37	43	−620	43
2	20.0	20.7	−17.0	18.7	−3	185	7	7
3	26.1	40.0	60.0	27.2	−53	−130	−4	−4
4	15.4	35.3	6.0	−17.0	−129	61	210	61
5	18.2	25.0	24.0	46.0	−37	−32	−153	−32
6	30.0	27.3	40.0	23.0	9	−33	23	23
7	26.1	22.2	22.2	27.3	15	15	−5	15
8	17.1	12.2	11.5	12.8	29	33	25	33
9	21.4	19.4	20.0	12.7	9	7	41	41
10	18.2	20.7	23.0	20.7	−14	−26	−14	−14
11	15.4	16.2	18.8	15.8	−5	−22	−3	−3
12	15.8	13.0	12.5	13.0	18	21	18	21
13	28.6	30.0	31.6	37.5	−5	−11	−31	−11
14	22.2	24.0	23.0	27.2	−8	−4	−22	−4
15	26.1	23.1	28.5	23.0	11	−9	12	12
16	21.4	19.4	20.0	18.8	9	7	12	9
17	20.7	21.4	20.7	20.7	−3	0	0	0
18	23.1	21.4	22.2	20.7	7	4	10	7
19	21.4	24.0	22.2	25.0	−12	−4	−17	−4
20	11.3	10.7	12.0	11.3	6	−6	0	0

## Data Availability

Not applicable.
